# Hand hygiene compliance and its associated factors among health care providers in primary hospitals of Waghimira Zone, Northeast Ethiopia: a mixed study design

**DOI:** 10.1186/s13756-022-01119-6

**Published:** 2022-05-31

**Authors:** Melese Alene, Dessalegn Tamiru, Getaw Walle Bazie, Wondwosen Mebratu, Natnael Kebede

**Affiliations:** 1 Health and Nutrition Project Management, Waghimira Field Office, Save the Children International, Waghimira Zone, Sekotta, Ethiopia; 2 Nutrition and Dietetics Department, Jimma University, Jimma, Ethiopia; 3 Department of Epidemiology and Biostatistics, School of Public Health, College of Medicine and Health Sciences, Wollo University, Dessie, Ethiopia; 4 Department of Health Promotion, School of Public Health, College of Medicine Health Sciences, Wollo University, Dessie, Ethiopia

**Keywords:** Hand hygiene, Compliance, Health care providers, Primary hospitals, Ethiopia

## Abstract

**Introduction:**

Hand hygiene compliance is the problem of developing nations particularly in Sub-Saharan Africa including Ethiopia. Despite a lot of efforts have been employed, healthcare-associated infections are the existing health care problems, leading to impaired quality of life, prolonged hospital stays, increased healthcare costs, morbidity and mortality. This study aimed to assess the magnitude and factors associated with hand hygiene compliance among health care providers working at the primary hospitals of Waghimira Zone, Northeast Ethiopia.

**Methods:**

Facility-based cross-sectional study design supplemented with qualitative research method was employed at the primary hospitals of Waghimira Zone from March 02–15, 2020. Simple random sampling using lottery method was applied to select 253 study participants. The data were coded on pre-arranged coding sheet and entered into Epi-Data version 3.1 and exported to SPSS version 25 for analysis. Descriptive statistics were displayed using tables and figures. Binary logistic regression analysis was used to test associations between the independent and the outcome variable. Multivariable logistic regression analysis was fitted to identify the independent predictors of hand-hygiene compliance at p-value < 0.05 and AOR with 95% confidence interval. Six Key Informant Interviews were conducted with purposively selected chief executive and clinical officers. Thematic content analysis was made and the findings were written sequentially with explanatory method.

**Results:**

One-fifth of the subjects (20.6%, 95% CI = 15.2, 24.9) had good hand hygiene compliance. Attended training on hand hygiene protocol (AOR = 3.18, 95% CI: 1.39, 7.28), accessible to adequate soap and water (AOR = 3.77, 95%CI: 1.52, 9.37), having alcohol for hand rub (AOR = 2.67, 95%CI: 1.18, 6.05) and having hand wash sink (AOR = 2.31, 95%CI: 1.03, 5.14) were significantly associated with hand hygiene compliance which also supported by the qualitative findings.

**Conclusions:**

Hand hygiene compliance among health care providers was low in the study area. Attended training on hand hygiene, accessibility to adequate soap and water, alcohol-based hand rub, and having hand washing sink in working area were statistically significant. Hence, the primary hospitals should be equipped with adequate supply to all the basic hand hygiene facilities.

## Introduction

Hand hygiene refers to an overall expression used to describe the appropriate use of hand washing with plain water (non-antimicrobial) and soap, especially the soap made from antiseptic agent which is used to minimize or limit the growth of microorganisms [1].

Since health care providers’ hands are the main usual mode of cross-transmission of different microorganisms, hand hygiene is the primary measure recognized to be effective in prevention of health care associated infections and the spread of antimicrobial resistance bacteria. About 50% of health care associated infections occurs due to poor hand hygiene practices of health care providers [2].

Across all settings, patients can acquire bloodstream infections, surgical site infections, urinary tract infections, chest/respiratory infections or gastrointestinal infections. Health-care workers are often the conduit for the spread of such infections to other patients in their care. It should also be noted here that many patients may carry microbes without any obvious signs or symptoms of an infection (colonized or sub clinically-infected). This clearly reinforces the need for hand hygiene, irrespective of the type of patient being cared for [3].

In Europe, health care associated infections cause 16 million extra-days of hospital stay, 37,000 attributable deaths, and contribute to an additional 110,000 deaths every year. Annual financial losses are estimated at approximately €7 billion, including direct costs only. In the USA, approximately 99 000 deaths were attributed to HCAI in 2002 and the annual economic impact was estimated at approximately US$ 6.5 billion in 2004. Information is again very scanty from low- and middle-income countries and no data are available at national or regional levels [3].

Healthcare-associated infections (HCAIs) are still one of the most challenging health care problems worldwide leading impaired quality of life, prolonged hospital stays, increased healthcare costs, mortality, and morbidity. Of the many factors associated with HCAIs, hand hygiene is considered the most effective, inexpensive, easily implemented measure for preventing HCAIs. [4].

The magnitude and scope of the HCAI burden worldwide appears to be very important and greatly underestimated in developing nations including Ethiopia [2]. Hand hygiene is the most effective, inexpensive measure for prevention of HCAIs and patient-to-patient transmission of microorganisms [5, 6].

Despite this truth on best of my understanding studies conducted in our country notably in Amhara region to measure its magnitude and varied associated factors are limited and there is no clear report on regarding this subject within the primary hospitals and this study aimed to fill this gap. The study also creates an opportunity to MOH, RHB and primary hospitals to see alternative strategies and initiatives in improving hand hygiene practices among health care providers.

The study can facilitate to spot doable action on factors and effects of inappropriate hand hygiene compliance and in turn will facilitate to develop ways to mitigate the matter or enhance the welfare of the patient by preventing cross transmission. Also, it will provide information for health care providers to take necessary precaution so as to prevent health care associated infections.

The finding of this study will also provide a baseline information for other researchers in doing further studies related to the topic, as well as it will provide acceptable recommendations, guidance and information for program planners, implementers, policy makers, and hence will enables them to come up with a feasible and convenient programmatic approach and initiatives to improve hand hygiene compliance in primary hospitals.

## Methods and materials

### Study area and design

A facility-based cross-sectional study design supplemented with qualitative research method was employed in Primary hospitals of Wagihmera Zone from March 02–15, 2020. Waghimra Zone is located 720 km away from Addis Ababa, the capital city of Addis Ababa and 435 km from Bahir Dar (the capital city of Amhara National Regional State). In this Zone, there are three primary hospitals located in Sekota, Dehana and Ziquala Districts.

### Population

The study populations were all health care providers who had contacts with patients during the time of data collection at the primary hospitals of Waghimira Zone. All health care providers who had a contact with patients, those who were actively working beyond six months in the facilities at the time of data collection were included in the study. The study populations for qualitative research were all purposively selected chief executive officers and clinical officers from the corresponding hospitals.

### Variables

The dependent variable was hand hygiene compliance. The independent variables were socio-demographic variables (age, sex, marital status, level of education, profession, and year(s) of working experience), type of working unit (Medical unit, Pediatric unit, Surgical unit, Intensive Care Unit, Laboratory Unit, Obstetrics and Gynecology Unit, OPD, Emergency Unit), hand hygiene knowledge and attitude related factors (level of knowledge on hand hygiene, level of attitude towards hand hygiene, knowledge on five moments of hand hygiene, training on hand hygiene protocols and guidelines), and availability and accessibility of hand hygiene facilities in patient wards (clean running tap water, hand washing soap, hand washing sink, alcohol-based hand rub, posters on hand hygiene, protocol and guidelines on hand hygiene, glove and knows availability of IP Committee).

### Sample size and sampling procedures

Primarily, the required sample was calculated using single population proportion formula based on the proportion of good hygiene practice 16.5% from the study conducted at Gondar University Hospital [7]. With the assumption of 5% margin of error (d), a standard Z score of 1.96 corresponding to 95% CI and 10% non-response rate, the sample size (n) was computed and obtained as 233.

However, the maximum sample size was obtained as 261 which was computed based on sample size calculation for the second specific objective (double population proportions formula) using EPI info statistical software version 7 by taking the proportion 9.7% of availability of hand towel/tissue paper from a study conducted in Gondar University Hospital, Amhara region, Ethiopia [7], and assuming power of 80%, 95% confidence level, the odds ratio of 1.90, and then sample size was adjusted.

With consideration of 10% non-response rate, finally, 261 was the total sample size for this study.

**Fig. 1:**
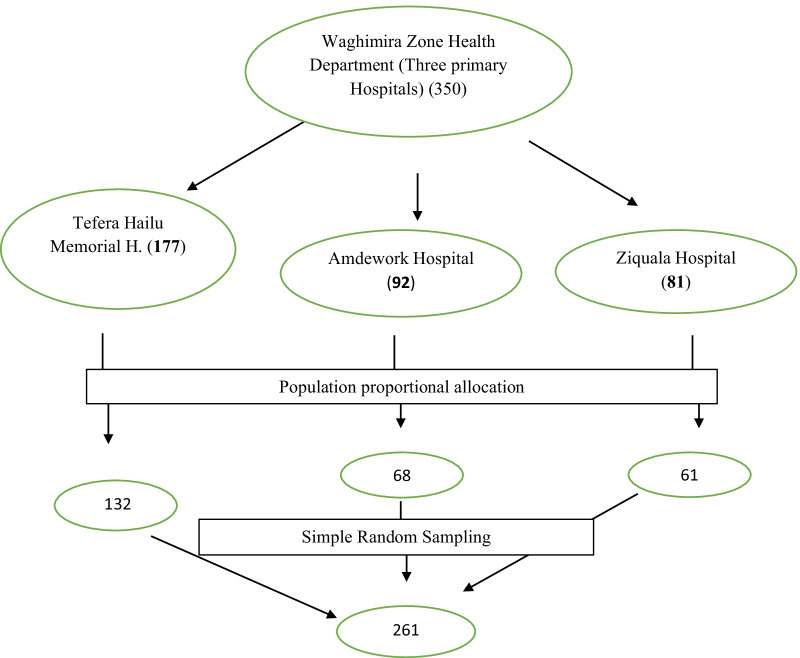
Sampling technique and procedure in primary hospitals of Waghimira Zone, 2020

Since the total number of primary hospitals were three in the zone, all of them were considered for this study. Prior to the actual survey, there were 350 health care providers actively working in the facilities with reference to the human resource units of the hospitals. Finally, with consideration of the proportional allocation to size, the study participants were recruited from each hospital using simple random sampling technique with lottery method (Fig. 1).

For qualitative study, six key informants of chief executive officers and clinical officers (medical directors) were purposively recruited from the corresponding hospitals and they were selected on the criterion that they did not participate in the quantitative study. Hence, the key informant interviews were conducted at the three sites parallel to the quantitative data collection period using a semi-structured interview guide.

### Data collection instruments and procedures

For quantitative study, data were collected through structured self-administered questionnaires. The tool was developed and adapted from standard sources such as WHO hand hygiene compliance questionnaires, observation checklists and by reviewing different literatures to address the research objectives. Data were collected by six BSc nurses trained on infection prevention and patient safety and the supervision was undertaken by three BSc Nurses who had a great deal of experience in data collection.

To assure the data quality, the data collection tool was pre-tested, and data collectors and supervisors were trained for one day. The questionnaire was first prepared in English. The English version questionnaire was translated to the Amharic version and translated back to English to maintain its consistency. It was pretested before the actual data collection days on 14 health care professionals who were working out of the study facilities at Sekota health center which has similar socio-demographic characteristics with the primary hospitals to check its clarity, reliability, consistency and the necessary modifications were made. Moreover, during data collection supervisors were responsible to check in the field how the data collectors were doing their task. The principal investigator was also closely supervising the field activity on a daily basis.

For qualitative study, six key informant interviews were conducted with chief clinical officer (medical directors) and chief executive officers who were selected purposively from those primary hospitals. Semi-structured guiding questions were prepared by reviewing to different literatures of qualitative researches. In order to get adequate data and reach to information saturation, note-taking supplemented with audio recording were employed during data collection. The data were collected with the principal investigator. There were two assistants with the investigator who took notes and observations during the discussion. In addition, a tape recorder was used to record the discussion to support the notes. The discussion was ceased when it reached saturation point.

### Data processing and analysis

Data were checked, cleaned and entered using Epi data statistical software version 3.1 and exported to SPSS version 25 for analysis. Knowledge of HCPs on hand hygiene compliance was assessed using twenty-five questions with either of two responses as Yes or No. The score for knowledge was graded as knowledgeable for those who scored the mean and above the mean value, otherwise it was considered as not knowledgeable. Attitude of HCPs towards hand hygiene compliance was measured using nine questions in which respondents were asked to choose a single option on a five point Likert scale where 1; strongly disagree and very heavy and 5; strongly agree and very easy. Final score for knowledge was calculated by adding up the points of knowledge questions and compute the mean value whereas attitude questions were analyzed with percent score (range 0–100). Attitude scores were graded assigning cut-off values used by similar studies high ≥ 75, moderate 50–74.9, poor < 50%.

Initially, the data were analyzed using bivariate analysis between the dependent variable (the hand hygiene compliance of health care professionals) and each of the factors potentially associated with the hand hygiene compliance. All variables having p-value < 0.25 in the bivariable analysis were further entered into the multivariable logistic regression model. Hence, the odds ratio with a 95% confidence level and the corresponding p-value was used to evaluate the association between independent variables with the outcome variable.

Qualitative data were analyzed manually using thematic content analysis where familiarization, coding, interpretation of the findings, and summarizing major themes from findings were done. The information was presented in narrations using well said word-by-word of the study participants as illustrations. Finally, the results were presented by supplementing with the quantitative findings sequentially using explanatory design.

### Ethical considerations

Ethical clearance was obtained from Institutional Review Committee of College of Medicine and Health Sciences, Wollo University. Permission letter to conduct the study was obtained from Waghimira Zone Health department and primary hospitals. Prior to data collection the general purpose of the research was provided to each of the participants with a local language then written and signed consent was obtained. All information that was obtained from the individual was treated confidential and the anonymity of the participants was kept by using only coding without mentioning names during interview. Each participant has the right to take out from the study without constraint. For this purpose, a one-page consent letter was attached as a cover page of each questionnaire stating about the general objective of the study and issues of confidentiality which was discussed by the data collectors before proceeding with the interview.

## Results

### Socio-demographic characteristics of the participants

A total of 253 study participants were included in this study and observed using observational checklist with 97% response rate. The age of the respondents ranged from 20 to 54 years with a mean age (± SD) of 28 ± 5.0 years. Among the respondents, 164 (64.8%) were in the age range of 25–34 years. The male health care providers accounted for 165 (65.2%) of the participants. Regarding profession, a higher proportion of respondents were nurses (115 (45.5%)) followed by health officers (37 (14.6%)). Large proportion of health care providers, 122 (48.2%) had a working experience of one to four years (Table 1).

**Table 1:** Socio-demographic Characteristic of study participants at primary hospitals of Waghimira Zone, North East Ethiopia, 2020 (n = 253)

Variable	Category	Frequency	Percent (%)
Age in years	18–24	60	23.7
	25–34	164	64.8
	≥ 35	29	11.5
Sex	Male	165	65.2
	Female	88	34.8
Profession	Physician	28	11.1
	Nurse	115	45.5
	Laboratory	23	9.1
	Health Officer	37	14.6
	Midwives	28	11.1
	Others*	22	8.7
Level of education	Diploma	84	33.2
	Bachelor	159	62.8
	2nd degree and above	10	4.0
Marital status	Married	104	41.1
	Single	144	56.9
	Divorced	05	2.0
Working experience	≤ 1 year	72	28.5
	1–4 years	122	48.2
	> 4 years	59	23.3
Unit of work	Medical ward	40	15.8
	Pediatric unit	16	6.3
	Surgical ward	35	13.8
	Intensive care unit	15	5.9
	Laboratory unit	22	8.7
	Obstetrics and gynecology unit	29	11.5
	OPD	42	16.6
	Emergency unit	26	10.3
	ART clinic	15	5.9
	Triage	13	5.1

*Integrated Surgery and Emergency Obstetrics, Radiography, Anesthesia, Clinical pharmacy, MSc, and Specialty

The respondents for qualitative study were described in terms of their socio-demographic characteristics that there were three chief executive officers and three clinical officers (medical doctors) purposively selected from each primary hospital parallel to the quantitative study. All of them were males.

### Hand hygiene compliance of health care providers

**Fig. 2:**
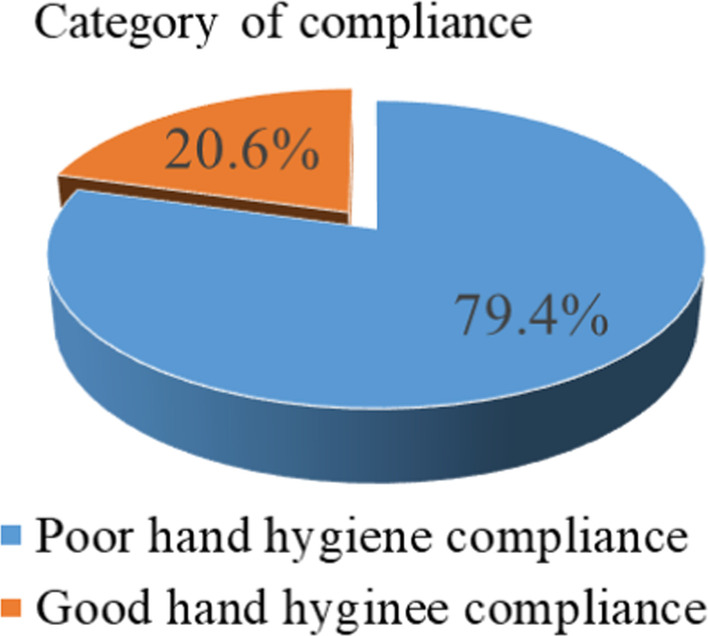
Hand hygiene compliance of health care providers at primary hospitals of Waghimira Zone, North East Ethiopia, 2020 (n = 253)

Over all, the hand hygiene compliance of health care professionals was 20.6% (95% CI = 15.2, 24.9) in the study area (Fig. 2). Regarding knowledge, more than half of the respondents (139 (54.9%)) of the respondents had knowledge on hand hygiene practice and 162 (64.0%) of the respondents knew that all the five moments of hand hygiene were important and critical. Additionally, seventy-seven (30.4%) of them reported that alcohol-based hand rub was available in their working area. Eighty-five (33.6%) of HCPs attended training on hand hygiene protocol in the last three years (Table 2).

**Table 2:** Variables related with hand hygiene compliance among health care providers in primary hospitals of Waghimira Zone, North East Ethiopia, 2020 (n = 253)

Variables	Category	Frequency	Percent (%)
Knowledge on hand hygiene	No	114	45.1
	Yes	139	54.9
Knows the 5 moments of hand hygiene	No	29	11.5
	Yes	224	88.5
Attitude towards hand hygiene	Poor	5	2.0
	Moderate	27	10.7
	High A	221	87.4
Received training on hand hygiene	No	168	66.4
	Yes	85	33.6
Knew the presence of IP committee	No	58	22.9
	Yes	195	77.1
HH promotion by hospital	No	92	36.4
	Yes	161	63.6
Available soap and water	No	168	66.4
	Yes	85	33.6
Available hand washing sink	No	153	60.5
	Yes	100	39.5
The presence of wall mount/individual ABHR	No	176	69.6
	Yes	77	30.4
Presence of gloves	No	91	36.0
	Yes	162	64.0
Presence of posters on hand hygiene	No	147	58.1
	Yes	106	41.9
Presence of protocol and guidelines	No	144	56.9
	Yes	109	43.1

### Factors associated with hand hygiene compliance

Variables that showed statistical significance at bivariate logistic regression were training attended on hand hygiene protocol, knowing the presence of infection prevention committee, promotion of hand hygiene practices, presence of adequate soap and water in the facility, availability of hand washing sink, availability of individual alcohol-based hand rub, and presence of poster on hand hygiene at working place.

In multivariable logistic regression analysis, training attended on hand hygiene protocol, presence of adequate soap and water, availability of hand washing sink, and availability of individual alcohol-based hand rub on their workplace were found to have a significant association with hand hygiene compliance among health care providers.

Health care providers who attended training on hand hygiene protocol were 3.18 times more likely to comply with guidelines and procedures of hand hygiene (AOR = 3.18, 95% CI: 1.39, 7.28) compared to those who did not attend training.

Findings from the qualitative study also indicated that training on hand hygiene has a great deal of contribution in prevention of cross transmission of microorganisms and can enhance the knowledge and practice of hand hygiene of health care providers. A 31-year male chief clinical officers commented that: *If there is consecutive training on hand hygiene, it can refresh the health care providers with the current guidelines and new procedures adopted from WHO and others and helps the HCPs to meet with the hand hygiene practices*.

Health care providers who were accessible to adequate water and soap at their working place were 3.77 times more likely to practice good hand hygiene (AOR = 3.77, 95% CI: 1.52, 9.37) as compared to health care workers who were not accessible to adequate water and soap.

An interview with key informants also indicated that lack of water and soap availability in their working areas has a significant contribution to health care provider’s compliance to hand hygiene. A 28-years male chief executive officer expressed about this situation as: *In our hospital, there is scarcity of water as a result of interruption of water supply. Additionally, this hospital is new and there is no enough budget to provide sufficient soap and other hygiene facilities*.

The odds of having good hand hygiene compliance were increased by 2.67 times among health care providers who got alcohol-based hand rub individually (AOR = 2.67, 95% CI: 1.18, 6.05) as compared with those who did not got alcohol-based hand rub individually.

Most participants depicted that absence of adequate hand hygiene facilities like alcohol-based hand rub at work place has significance influence on health care providers not to comply with WHO hand hygiene guidelines and procedures. A 33-years male chief executive officers at hospitals noted that: *For hand disinfection, health care provider need to get enough supply of alcohol-based hand rub in the hospital*.

Health care providers who had hand washing sink in their working place were 2.31 times more likely to practice good hand hygiene (AOR = 2.31, 95% CI: 1.03, 5.14) compared with those who were not accessible at their working place.

An interview with chief clinical officer also showed that there was a need of maintaining enabling environment like availing sink in the ward. A 25-years male chief clinical officer commented that: *As to me, there is a lot of problems related to accessibility hand washing facilities like sinks in some wards including OPD and emergency wards. During construction of buildings, sanitary facility design was not considered* (Table 3).

**Table 3:** Factors associated with hand hygiene compliance among health care providers of in primary hospitals of Waghimira Zone, North East Ethiopia, 2020 (n = 253)

Variables	Categories	Hand hygiene compliance		COR (95% CI)	AOR (95% CI)
		Good	Poor		
Knew the presence of IP committee	Yes	46	149	2.68(1.08, 6.63)	1.14(0.36, 3.65)
	No	6	52	1	1
Attended hand hygiene training	Yes	28	57	2.95 (1.58, 5.51)	3.18(1.39, 7.28)*
	No	24	144	1	**1**
Promotion of hand hygiene by hospital	Yes	41	120	2.52(1.22, 5.18)	0.96(0.35, 2.59)
	No	11	88	1	1
Adequate soap & water in the hospital	Yes	29	56	3.27(1.74, 6.12)	3.77(1.52, 9.37)*
	No	23	145	1	**1**
Hand washing sink is adequate and accessible	Yes	27	73	1.89(1.02, 3.50)	2.31(1.03, 5.14)*
	No	25	128	1	**1**
Alcohol based hand rub is available	Yes	27	50	3.62 (1.74, 6.13)	2.67(1.18, 6.05)*
	No	25	151	1	**1**
Presence of posters on hand hygiene	Yes	31	75	2.48(1.33, 4.63)	2.53(0.85, 7.52)
	No	21	126	1	1
Availability of protocol and guideline on hand hygiene	Yes	28	81	1.73(0.94, 3.19)	0.87(0.30, 2.54)
	No	24	120	1	1

*COR* Crude odds ratio, *AOR* adjusted odds ratio, *CI* confidence interval

NB: *significant at 5% significance level shown in bold

## Discussion

Hand hygiene is the primary action to prevent health care-associated infection and reduce the spread of multi-resistant organisms. Adherence of HCPs to recommended hand hygiene procedures has been unacceptably poor [8]. Poor adherence of HCPs to hand hygiene and its complication has impact on the patients and health care settings [2]. This study aimed to assess hand hygiene compliance of health care providers and associated factors in primary hospitals of Waghimira Zone, Northeast Ethiopia. Therefore, this study showed that one fifth (20.6%) of health care providers had a good hand hygiene compliance. This finding is consistent with a study conducted in Debre-Birhan referral hospital of Ethiopia, which was 22% that they followed WHO standards and guidelines of hand hygiene. However, this is lower than studies conducted in India (35%), Vetnam (39%), Sub-Saharan countries (68.9%) and Black lion Specialized referral hospital of Ethiopia (79%) [9–13]. This might be due to the presence of not actively working Infection Prevention and Control Committee (IPCCs) to each facility during the survey, inaccessibility of hand hygiene resources in Waghimira Zone primary hospitals, and there might be a lack of knowledge about Hand Hygiene practice, and poor hand hygiene promotion in the hospitals.

Health care providers who attended training on hand hygiene protocol and guideline were more likely practicing hand hygiene compliance than health care professionals who did not ever attended training. This result is consistent with studies from UK and India which showed that training had positive relationship with hand hygiene compliance in all medical staff [14, 15]. This might be because of the fact that training could build the knowledge and commitment of health care providers which had a significant association with hand hygiene compliance. The other reason might be due to an expectation that health care professionals who got training on this skill are expected to be a role models for others in terms of practicing good HH, and Knowledge of HCPs will help identify risk and benefit of practicing on the ways of prevention of health care associated infections transmission.

Health care professionals who were adequately accessible to soap and water for hand washing in their work place were more likely to had good hand hygiene compliance as compared to health care professionals who did not get adequate soap and water. This finding is similar with the studies done at Black Lion hospital, Ethiopia, but the finding from Gondar university teaching hospital, Ethiopia did not revealed a significant relation between having adequate soap and water in the working place and hand hygiene compliance [7, 13]. This may be due to difference in hospitals setting, availability and accessibility of hand washing facilities, delay in procurement process and budget inadequacy.

The odds of having individual alcohol-based hand rub in their work place appeared to be associated with hand hygiene compliance of health care providers which is consistent with studies done in Taiwan and Brazil and in Gondar university teaching hospital, Ethiopia [7, 16, 17]. This might be due to the fact that the presence of alcohol-based hand rub at point of care can stimulate the HCPs to practice hand hygiene compliance and inaccessibility of hygiene related resources in their nearby ward might be one of the reasons for not exercising hand hygiene recommendations.

Health care professionals who had adequate and accessible to hand washing sink were more likely had good hand hygiene compliance compared to health professionals who were not adequately accessible to sink for hand washing. However, study conducted at Gondar University teaching hospital showed no significant association between adequate soap and water and hand hygiene compliance this may be due to the presence of improved infrastructure found in the hospital [7].

## Conclusions

Hand hygiene compliance was found to be low among HCPs in the primary hospitals of Waghimira Zone. Attended training on hand hygiene protocol, presence of adequate soap and water, availability of hand washing sink, and availability of individual alcohol-based hand rub on their work place were found to be the determinants of hand hygiene compliance among health care professionals. Therefore, all the primary hospitals should be equipped with adequate knowledge and skills on hand hygiene, available and accessible to all the options of basic hand hygiene facilities.

## Data Availability

The datasets used and/or analysed during the current study are available from the corresponding author on reasonable request.

## Abbreviations

AOR: Adjusted odds ratio
COR: Crude odds ratio
HCAI: Health care associated infections
HCPs: Health care providers
IP: Infection prevention
KIIs: Key informant interviews
MCH: Maternal and child health
NGO: Non-governmental Organization
OPD: Out-patient department
SD: Standard Deviation
SPSS: Statistical package for social sciences
WHO: World Health Organization
